# Island water stress: analyzing the Canary Islands’ hydrological response to climate change

**DOI:** 10.1007/s10661-026-15219-y

**Published:** 2026-04-10

**Authors:** Juan C. Santamarta, Alejandro García-Gil, Susana Clavijo-Núñez, Noelia Cruz-Pérez

**Affiliations:** 1 Departamento de Ingeniería Agraria y del Medio Natural, Universidad de La Laguna (ULL), Tenerife, Spain; 2 Geological and Mining Institute of Spain (IGME), Spanish National Research Council (CSIC), Calle Ríos Rosas 23, 28003 Madrid, Spain

**Keywords:** Hydrological cycle, Freshwater availability, Sustainable water use, Hydrological modeling

## Abstract

Climate change and human activities threaten water resources in the Canary Islands, where increasing temperatures and decreasing precipitation intensify water stress. In order to develop strategies to protect water resources, it is necessary to know how water availability will evolve in this region. The *Fundación para la Investigación del Clima* (FICLIMA) methodology offers high-resolution projections (100 m × 100 m), which allow us to understand how climate change alters the long-term dynamics of the water balance in islands with complex orography and microclimates. The results reveal a decrease in the water balance due to increased evapotranspiration and stable or reduced precipitation. Severe decreases in the water balance are expected by the end of the century, reaching reductions of 50–75% on El Hierro or total depletion on Gran Canaria. Sustainable water management and the implementation of adaptive policies will be essential to guaranteeing water security in the future.

## Introduction

Water is fundamental to all life on Earth, serving as the cornerstone of ecosystems, agricultural production, industrial processes, and overall human well-being. However, the availability of this vital resource is under increasing pressure due to global changes, including population growth, socioeconomic changes, land use alterations, and, of course, climate change (Gudmundsson et al., 2016). These interconnected drivers significantly influence both water supply and demand, threatening the balance required to sustain natural systems and human societies (Kristvik et al., 2018). Future projections suggest that these pressures will only intensify, resulting in more regions facing severe water stress (Alcamo et al., 2007). This is especially true and problematic for areas already struggling with water scarcity, which includes the Canary Islands (Munia et al., 2020; Vicente-Serrano et al., 2016).

Recognizing the severity of this issue, governments, institutions, and organizations worldwide have developed strategies and policies to support sustainable water management and safeguard water resources. Diverse approaches to address water challenges are being adopted, including improving infrastructure for water storage and distribution, implementing policies for efficient water use, and advancing research into climate-resilient water management practices (World Water Council, 2018). In general, these approaches can be divided into two categories. The first focuses on increasing water supply by water reuse, desalination of seawater, enhancing surface and groundwater storage infrastructure, and so on. The other category is demand oriented and aims to reduce water consumption and improve water productivity. These approaches include enhancing water-use efficiency for domestic, industrial, and agricultural use and minimizing water losses in transport and distribution systems (Cosgrove & Loucks, 2015; Scheele & Malz, 2007).

No matter if the measures are supply or demand oriented, they should be based on evidence (UNESCO, 2022), through analyses of current and projected water demand, surface and groundwater availability, and future climate change scenarios (Menció et al., 2010). But first, it is essential to understand the dynamics of the hydrological cycle and water balance (WB), including their components and all of the factors that influence them.

The hydrological cycle encompasses the continuous movement and exchange of water within the earth and atmosphere (Wang et al., 2021), involving key components such as precipitation, interception, evapotranspiration, infiltration, and runoff (Mohajerani et al., 2021). Among these processes, precipitation (in either liquid or solid state) acts as the primary input of the natural water balance (Balasubramanian & Nagaraju, 2015), delivering water to the surface. Interception by vegetation influences the amount of water that reaches the soil, and infiltration further directs water into underground reservoirs, recharging aquifers. Outputs include processes like evapotranspiration (ETo), where water is transferred back to the atmosphere from soil and vegetation, and runoff, which transports water away from the land surface, contributing to rivers, lakes, and oceans (AGW-Net et al., 2015; Easton & Bock, 2015). These components interact dynamically across temporal and spatial scales and are shaped by region-specific factors such as topography, soil properties, vegetation cover, and climate regimes (Suliman et al., 2024).

The above-mentioned hydrological processes are strongly affected by human actions, such as water abstraction (for agriculture, domestic, and industrial use), water return and water transfer, land use alterations, urbanization, deforestation, and flow regulation (Bosmans et al., 2017; European Commission, 2015). Additionally, human activities contribute to the increase in greenhouse gases, driving climate change, which also has a profound impact on the hydrological cycle and water availability (Deng et al., 2023). Climate change leads to alterations in precipitation patterns (spatiotemporal distribution and amount), which directly affect the volume of water input into the hydrological system at specific locations (Trenberth, 2011). Rising temperatures further exacerbate these effects by increasing evapotranspiration rates, thereby intensifying water loss from soil and vegetation (European Commission,  2015).

By incorporating these anthropogenic components into the natural hydrological cycle, the water balance can be calculated (European Commission, 2015), providing an essential estimation of water availability of a specific hydrological unit. The evaluation of water balance therefore involves the analysis and quantification of (1) hydrological components, including runoff, precipitation, and actual and potential evapotranspiration, using available climate data, and (2) estimation of human activities such as water abstraction and return (Menció et al., 2010). These evaluations are crucial in accounting for all water flowing into and out of a reference area, understanding the effects of climate variability and human activities on natural water resources, and identifying water stress conditions.

In order to analyze the hydrological impacts caused by climate change, climate model projections are used. Although these models make it possible to predict how the water balance will evolve in the coming years, they carry associated uncertainty, especially in relation to the calculation of precipitation (Her et al., 2019). Generally, the uncertainty is related to the choice of general circulation models (GCMs), representative concentration pathways (RCPs), and hydrological models (Krysanova & Hattermann, 2017).

Previous studies have also identified the importance of developing regional models to analyze long-term hydrological dynamics versus global models (Krysanova & Hattermann, 2017). These types of models can provide valuable information when analyzed over long time series, allowing us to understand the type of change that different variables undergo (Dey & Mishra, 2017).

This article evaluates the potential impacts of human actions and climate change on hydrological processes and water balance in the Canary Islands. More specifically, it focuses on how climate change alters long-term water balance dynamics and the spatiotemporal distribution of water resources. To this end, climate projections have been generated based on models from the Sixth Assessment Report (AR6) of the Intergovernmental Panel on Climate Change (IPCC), using experiments from the Coupled Model Intercomparison Project Phase 6 (CMIP6), a 30-year reference period. The novelty of the article lies in the ability to adapt the FICLIMA method to the conditions of the archipelago in order to analyze high-resolution climate projections (resolution 100 × 100 m) of water balance, taking into account climate fluctuations in isolated ecosystems.

Such insights are critical for understanding future trends in water availability, enabling policymakers and water managers to devise adaptive and sustainable strategies to secure potable water supplies for residents and support the region’s socioeconomic activities. This is especially important for the Canary Islands, since the region highly depends on tourism and agriculture, which are two of the most water-demanding sectors.

The paper is structured as follows: after this introduction, the study area is presented, with a focus on the water balance, water management, and associated challenges in the Canary Islands. The methodology of the research is described in the, followed by the results and discussion in the “Results and discussion” section. The final section provides conclusions, summarizing the main findings of the paper.

### Study area

**Fig. 1:**
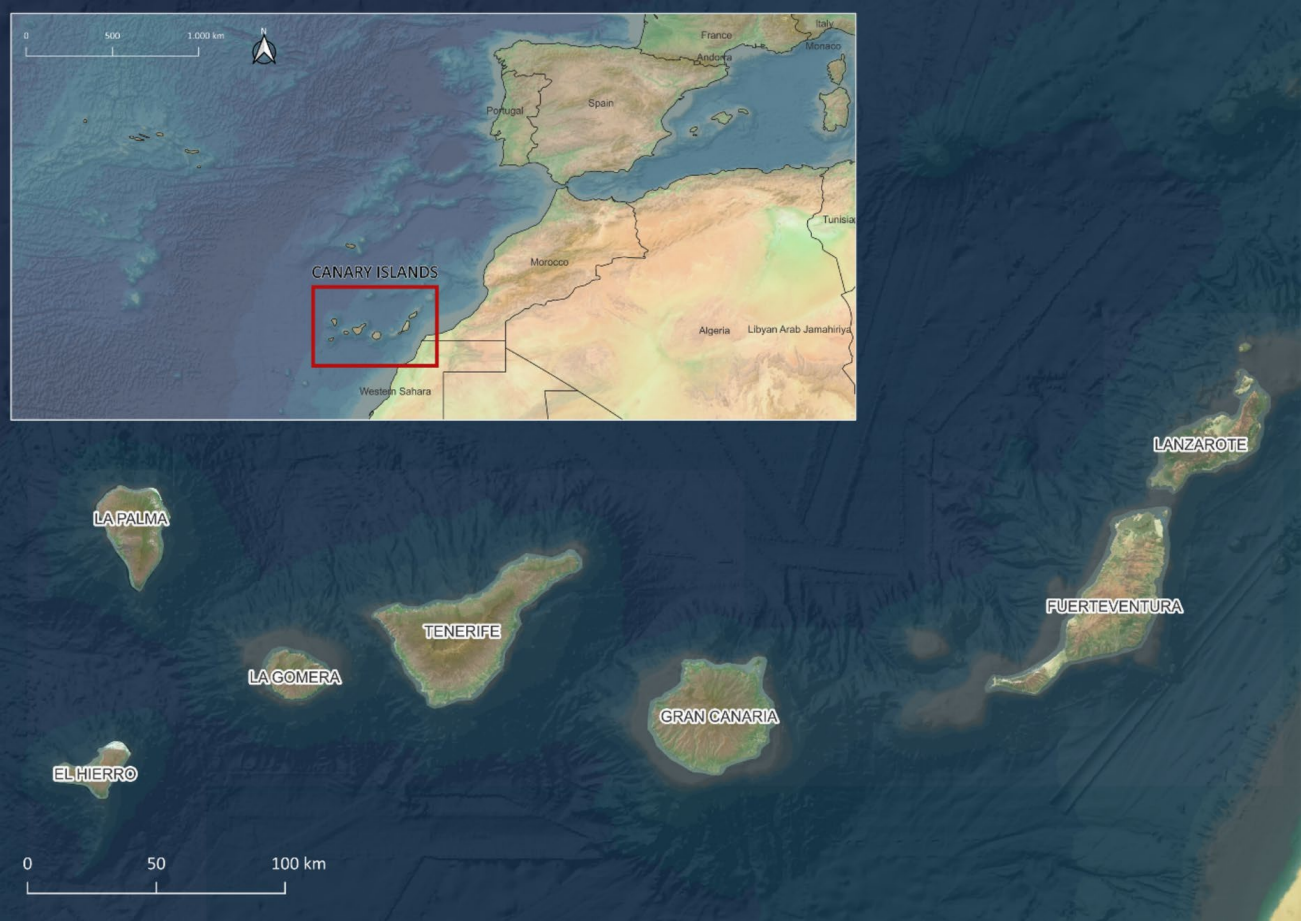
Location of the case study: Canary Islands (Spain)

The Canary Islands are a volcanic archipelago composed of seven main islands, namely, El Hierro, La Palma, La Gomera, Tenerife, Gran Canaria, Fuerteventura, and Lanzarote (Fig. 1). Located in the Atlantic Ocean, approximately 100 km off the northwest coast of Africa, they are Spanish territory and Europe’s outermost region. The total area of the Islands is nearly 7500 km^2^, with Tenerife being the largest island (1929 km^2^) and El Hierro being the smallest (224 km^2^). The archipelago is known for its complex orography, increasing from east to west, with topography extending up to 3715 m above sea level (Mount Teide).

The islands are situated in a subtropical climate zone (Herrera et al., 2001), with annual temperatures around 18 °C to 24 °C and average annual rainfall of approximately 400 mm (Weather & Climate, 2024). According to the aridity index, which is an indicator of potential water availability (Zarch et al., 2015), more than 70% of the Canary Islands are classified as arid or semi-arid (Corral Quintana et al., 2015). The eastern islands, Lanzarote and Fuerteventura, are especially arid due to their proximity to the Sahara Desert, while the western islands display a wider range of climates, though arid and semi-arid conditions are still predominant in most of the islands (Luque-Söllheim et al., 2024).

The water balance between water demand and supply and, therefore, water availability in the Canary Islands is under increasing pressure due to unique geographical, climatic, and socio-economic conditions of the region.

On the supply side, the Canary Islands depend heavily on groundwater, which accounts for nearly 80% of the water supply and is mostly extracted from privately owned aquifers. This is especially true for the western islands. The eastern islands, however, depend strongly on seawater desalination, since underground water sources are very limited (Santamarta et al., 2022). Desalination plants are crucial for meeting the water demands of the Canary Islands. However, the process requires a significant amount of energy, contributing to higher carbon emissions (Cruz‐Pérez et al., 2022; León et al., 2021). Seawater desalination accounts for approximately 77% of the archipelago’s desalinated water supply and has the highest carbon footprint among the region’s water production methods (Cruz‐Pérez et al., 2022; León et al., 2021). To mitigate its environmental impact, researchers are exploring the integration of renewable energy sources (Gils & Simon, 2017; Peñate et al., 2011; Velázquez-Medina, 2023).

The agricultural, urban water supply, and tourism sectors exhibit the highest levels of gross water demand in the archipelago (Santamarta et al., 2014). Although the agricultural sector accounts for nearly 50% of total water consumption, an increasing trend in urban and tourism-related water use has been observed in recent decades (Schmitz et al., 2017; Weiser et al., 2021; Cruz‐Pérez et al., 2023). For example, water consumption associated with tourism accounts for approximately 12% of total water demand on the island of Gran Canaria (Herández-Martín & León, 2024). In addition, population growth and a high percentage of water losses during transport and distribution put further pressure on water availability in the region.

These challenges are intensified by the impacts of climate change, including decreased precipitation and rising temperatures (Carrillo et al., 2023; Expósito et al., 2015). These shifts lead to greater aridity and amplify water scarcity across the region, creating an urgent need for sustainable and adaptive water management strategies.

To address the above-mentioned issues, and in alignment with the European Union’s Water Framework Directive, the Canary Islands have implemented comprehensive hydrological planning through Island Hydrological Plans, which serve as the cornerstone of water resource management. These plans, revised every 6 years, examine the hydrological status and future trends of each island (Santamarta et al., 2022). Their primary goal is to ensure that current and future water supply meet the needs of society (regarding both quantity and quality).

Therefore, water governance structures and regulatory frameworks play a key role in facilitating effective water management and conservation efforts in the Canary Islands (Rodríguez-Urrego et al., 2022). The Canarian Law of Water 12/1990 granted each island autonomy over its hydrological system, allowing management, organization, and planning through its own Insular Water Council (Rodríguez-Urrego et al., 2022). To address the impacts of climate change on water resources, these policies should integrate climate adaptation strategies (Schmitz et al., 2017).

For these plans to be as effective as possible, continuous monitoring and analysis of water resources are essential to adapting to future changes. This article contributes to this endeavor by providing insights into the hydrological processes and trends in the Canary Islands.

## Methodology

This study employs the FICLIMA approach developed by Ribalaygua et al. (2013), which integrates large-scale climate simulations with regional hydrology. FICLIMA assesses potential changes in water availability across three future timeframes: the near future (2021–2050), the mid-future (2041–2070), and the late future (2071–2100). It should be noted that the water balance, as well as the indicators necessary for its calculation, has been based on two direct indicators: temperature and precipitation. The methodology has been structured in three phases.

### Weather information

Historical climate records from 1985 to 2014 serve as a baseline for comparison. Through meteorological observations and climatic information on temperature and precipitation variables, a historical data for the calculation of the water balance has been created.

Meteorological data for the Canary Islands, particularly temperature and precipitation records, were compiled to develop a long-term climate database (ideally spanning 30 years or containing at least 2000 records). Data were sourced from various weather stations across the archipelago. Complementary climatic information was also utilized to address gaps in direct observations, ensuring broader spatial coverage through the use of Geographic Information Systems (GIS), reanalysis datasets, and climate models.

Using GIS techniques, multiple climate data layers were extracted, including historical and geographical information from the CanaryClim database (Patiño et al., 2023), as well as GIS layers related to land use that incorporate variables such as aspect and land surface temperature. Additionally, climate data from the SITCAN Canary Islands Atlas were included, comprising layers of precipitation, temperature, and cloud cover (Luque-Söllheim et al., 2024). The extraction of different layers has been essential in order to take into account the orographically complex locations, such as northness or eastness, as well as the presence of microclimates. The main limitation of this step is associated with the accessibility of the data collected. However, collaboration with other entities, such as the University of Las Palmas de Gran Canaria and Teide National Park, has made it possible to compile all these sources.

Reanalysis data, specifically ERA5, were employed to estimate atmospheric and surface variables from 1950 onward, offering hourly data on factors such as relative humidity and precipitation at multiple altitudes. ERA5 provides global coverage encompassing both oceanic and coastal regions, with a spatial resolution of 0.25° × 0.25°. To achieve a more detailed representation of terrestrial processes, specific variables were additionally drawn from ERA5-Land, which offers enhanced horizontal resolution (0.1° × 0.1°) over continental areas.

**Fig. 2:**
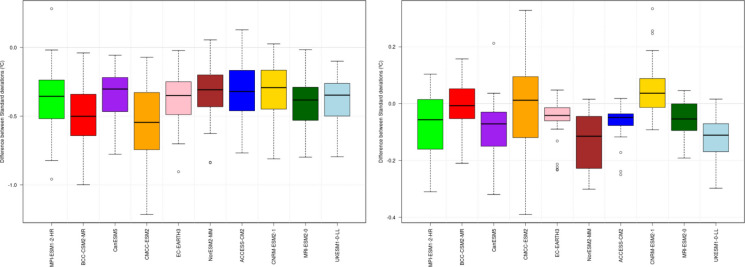
Validation results of difference between standard deviation (SD) statistic to maximum temperature values before (left) and after (right) the correction of the 10 CMIP6 models considered

**Fig. 3:**
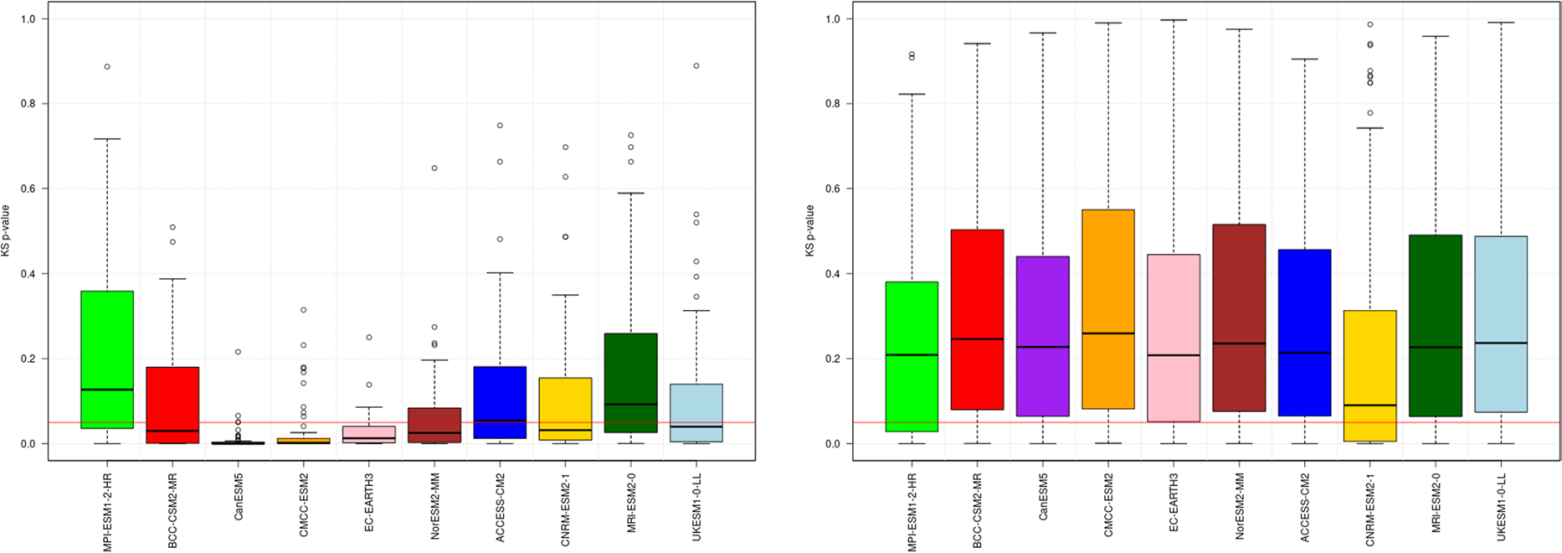
Validation results for precipitation of the application of the Kolmogorov–Smirnov (KS) test values before (left) and after (right) the correction of the 10 CMIP6 models considered

Additionally, the study incorporated CMIP6 climate models aligned with the IPCC AR6 and the latest shared socioeconomic pathways (SSPs), enhancing the analytical framework for future climate scenarios in the region. An ensemble of ten CMIP6 models was selected to capture uncertainty in climate projections (Table 1). These models underwent evaluation, leading to the exclusion of one precipitation and one temperature model. Model evaluation relies on the bias of the mean and standard deviation, computed as the average total error at each station, as these metrics reflect how well spatial climate patterns are reproduced. Large bias dispersion among observatories may indicate distorted regional variability and unrealistic representation of key climatic features. Beyond mean conditions, climate variability is assessed using the non-parametric Kolmogorov–Smirnov test, which compares entire probability distributions. Figure 2 presents, as an example, the results of the standard deviation (SD) validation for the temperature variable on the island of Gran Canaria. Figure 3 shows, for the same island, the results of the Kolmogorov–Smirnov (KS) test applied to the precipitation variable.

**Table 1:** CMIP6 models used for each study variable

Models	Temperature	Precipitation
*ACCESO-CM2*	Yes	Yes
*BCC-CSM2-MR*	Yes	Yes
*CanESM5*	Yes	No
*CMCC-ESM2*	No	Yes
*CNRM-ESM2-1*	Yes	Yes
*EC-EARTH3*	Yes	Yes
*MPI-ESM1-2-HR*	Yes	Yes
*MRI-ESM2-0*	Yes	Yes
*NorESM2-MM*	Yes	Yes
*UKESM1-0-LL*	Yes	Yes

### FICLIMA methodology in the Canary Islands

To adapt the FICLIMA methodology to the Canary Islands, the spatial climate patterns from 1985 to 2014 were analyzed. A geographically weighted regression (GWR) was employed to integrate topographic and climatic variables with observational data, enhancing the extrapolation of local climate conditions across the islands. For each island, 25 tests were conducted on key variables maximum and minimum temperature and precipitation (Table 2). The results were validated through statistical, spatial, and visual assessments at annual, monthly, and seasonal scales.

**Table 2:** Selection of layers for the spatial characterization of each of the Canary Island climate distributions

Island	Climate variable	Topographic and climate layers used
La Palma	Maximum temperature	Altitude, distance to seashore, aspect, northness, eastness, cloudiness
	Minimum temperature	Altitude, distance to seashore, aspect, northness, eastness, cloudiness
	Precipitation	Altitude, distance to seashore, northness, eastness, cloudiness, LST, NDVI
El Hierro	Maximum temperature	Altitude, distance to seashore, aspect, cloudiness
	Minimum temperature	Altitude, distance to seashore, aspect, cloudiness
	Precipitation	Altitude, aspect, northness, eastness, cloudiness
La Gomera	Maximum temperature	Altitude, aspect, northness, cloudiness
	Minimum temperature	Altitude, aspect, northness, cloudiness
	Precipitation	Altitude, aspect, northness, cloudiness
Tenerife	Maximum temperature	Altitude, aspect, northness, cloudiness
	Minimum temperature	Altitude, aspect, northness, cloudiness
	Precipitation	Altitude, aspect, northness, cloudiness
Gran Canaria	Maximum temperature	Altitude, distance to seashore, aspect, northness, cloudiness
	Minimum temperature	Altitude, distance to seashore, aspect, northness, cloudiness
	Precipitation	Altitude, distance to seashore, aspect, northness, cloudiness
Fuerteventura	Maximum temperature	Altitude, cloudiness
	Minimum temperature	Altitude, cloudiness
	Precipitation	Altitude, distance to seashore, northness, eastness, cloudiness
Lanzarote	Maximum temperature	Altitude, distance to seashore, northness, eastness, cloudiness
	Minimum temperature	Altitude, distance to seashore, northness, eastness, cloudiness
	Precipitation	Altitude, aspect, cloudiness

### Water balance calculation

Using previous historical records, the climate data reference is established. Climate models were then downscaled using FICLIMA, which employs analogue stratification. At this stage, days characterized by overarching atmospheric patterns (predictors) that resemble locally observed records (predictands) are identified. The underlying assumption of the method is that analogous atmospheric conditions produce comparable local impacts. To operationalize this, the approach applies a weighting of Euclidean distances among analogs across three synoptic windows and four large-scale fields, thereby accounting for nonlinear relationships between atmospheric circulation and local responses.

The second stage for the precipitation variable involves identifying the “problem” days and selecting the most comparable past days as determined in the previous step. An initial precipitation estimate is then derived by averaging the values of these analogous days. Subsequently, the values are ordered within their distribution, and the actual precipitation is assigned according to this ranked distribution.

Calculation of the monthly water balance was developed in a simplified form, as a function of precipitation and evapotranspiration (Eq. 1). Thus, if the soil loses its entire reserve, the WB takes the value of 0. Additionally, regression adjustments refine precipitation estimates by generating daily rainfall values based on historical analogues. The study also simulates extreme precipitation events, which are critical for understanding the impacts of droughts and floods on the islands’ water balance. By accounting for both timing and magnitude, FICLIMA enhances the accuracy of rainfall projections. Finally, validation processes address uncertainties inherent in climate projections, ensuring that the models remain aligned with the Canary Islands’ diverse terrain and microclimates.

1 $$WB{}_m\;=WB{}_{m-}{}_1+P_{r{}_m-}ET_{o_m}$$

*WB*_*m*_ represents the water balance for a given month (*m*), while *Pr*_*m*_ and *ETo*_*m*_ correspond to the total precipitation and reference evapotranspiration for the same period, respectively. Additionally, *WB*_*m*−1_ denotes the water balance recorded in the preceding month.

This methodology is particularly suited to the Canary Islands, where unique weather patterns and evolving water demands present ongoing challenges. It captures microclimatic variations by assessing rainfall changes from coastal areas to higher elevations, identifies extreme weather patterns such as prolonged droughts or intense rainfall that threaten water resources, and provides insights for future water management strategies. By integrating advanced climate modeling techniques, this approach enables stakeholders to anticipate and mitigate the effects of climate change on the region’s water balance, ensuring the long-term sustainability and resilience of the Canary Islands’ water systems.

## Results and discussion

The methodology made it possible to analyze the evolution of water resources in the Canary Islands, which should be protected and studied. For the calculation of the different predictions, nine IPCC AR6 models and 4 climate scenarios have been used.

**Fig. 4:**
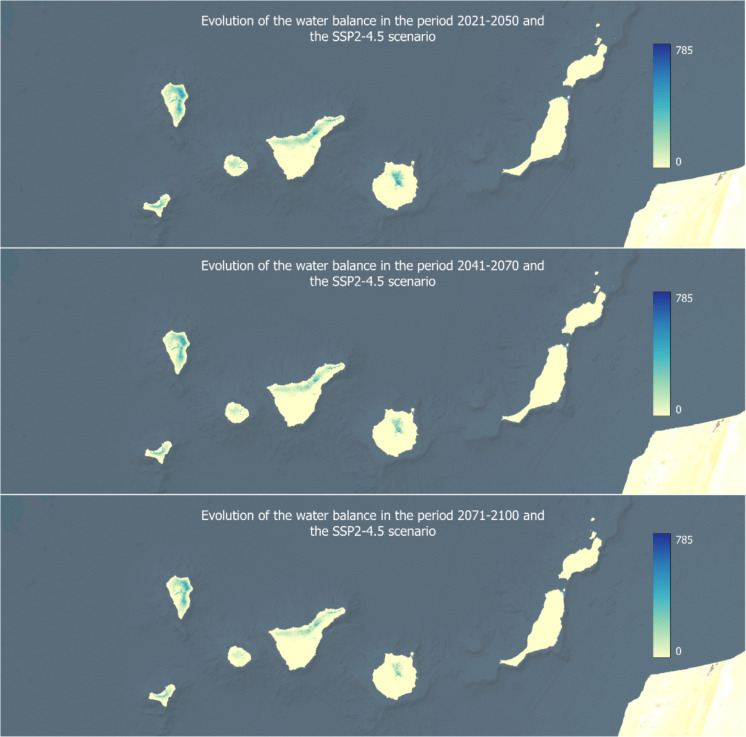
Evolution of the water balance in three time periods for the SSP2-4.5 scenario

**Fig. 5:**
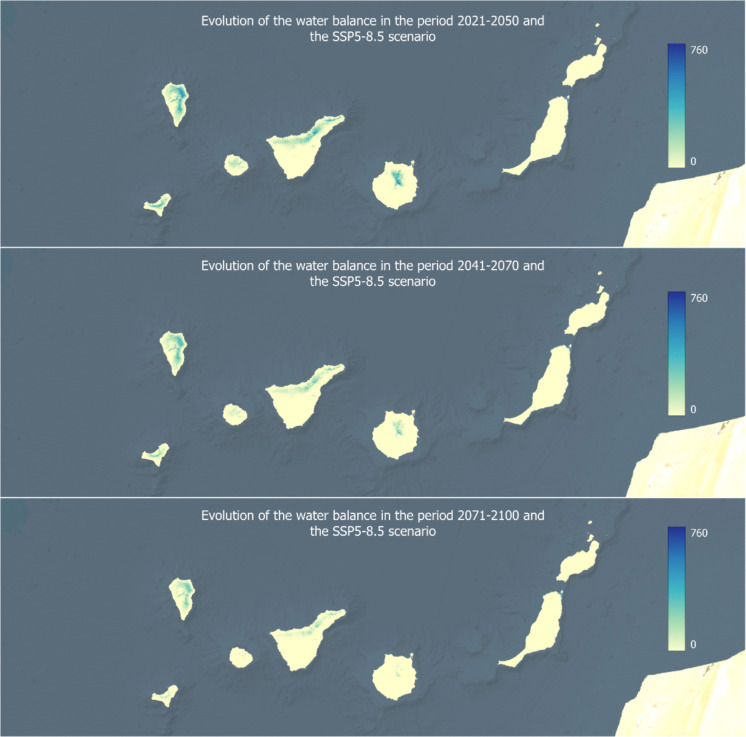
Evolution of the water balance in three time periods for the SSP5-8.5 scenario

Using the different data layers available on the public access platform SICMA-Canarias (Santamarta et al., 2025), the water balance in the Canary Islands is represented in Figs. 4 and 5. These specify the results of the water balance across three future timeframes, using the median of the models, for an intermediate climate scenario (SSP2-4.5) and a worst-case scenario (SSP5-8.5). Although the calculations have been developed for the four shared socioeconomic pathways (SSPs), only these two scenarios are included as a representation. In this way, a regional study is presented to show the differences in the evolution of the water balance in different locations.

The results obtained on the water balance in each of the islands of the Canary archipelago are presented below.

- La Palma: Historical data reveals that only the humid areas of La Palma, particularly the eastern, northern, and northeastern regions, maintain a significant amount of water throughout the year. In the short and medium term, a slight increase in water availability is projected for the northeast of the island. However, long-term predictions indicate a decline in available water due to an increase in evapotranspiration (ETo), while precipitation remains stable or decreases.
- El Hierro: The annual water balance in El Hierro shows that only moderate amounts of water remain available along the cliffs in the center of the island, approximately 200 mm, with smaller amounts in the northeastern plateau, barely reaching 100 mm. Future projections suggest a reduction in the water balance by 50 to 75%, with a decline of up to −150 mm in the central region.
- La Gomera: The annual water balance in La Gomera indicates small amounts of available water in the central highlands and mid-altitude regions, such as Garajonay National Park located in the center of the island. Due to persistent cloud cover for most of the year, this area maintains an average of around 100 mm. Future projections suggest a partial to near-total reduction in the water balance.
- Tenerife: Analysis of historical and annual data in Tenerife highlights that only the northern parts of the island show small amounts of available water, with maximum values around 200 mm in a natural park in the north. Future predictions estimate reductions of approximately 50% in the water balance, translating to decreases of 50 to 100 mm.
- Gran Canaria: The annual water balance in Gran Canaria reveals that only small amounts of water remain in the mountainous northern and central regions of the island, with barely 100 mm. Future projections estimate an almost complete depletion of the island’s water reserves.
- Fuerteventura and Lanzarote: For the easternmost islands, Fuerteventura and Lanzarote, the annual water balance is effectively zero, meaning that no significant precipitation allows for water availability at any time of the year. The overall trend for these islands, characterized by rising temperatures and decreasing precipitation, will only exacerbate water stress in Fuerteventura and Lanzarote.

**Fig. 6:**
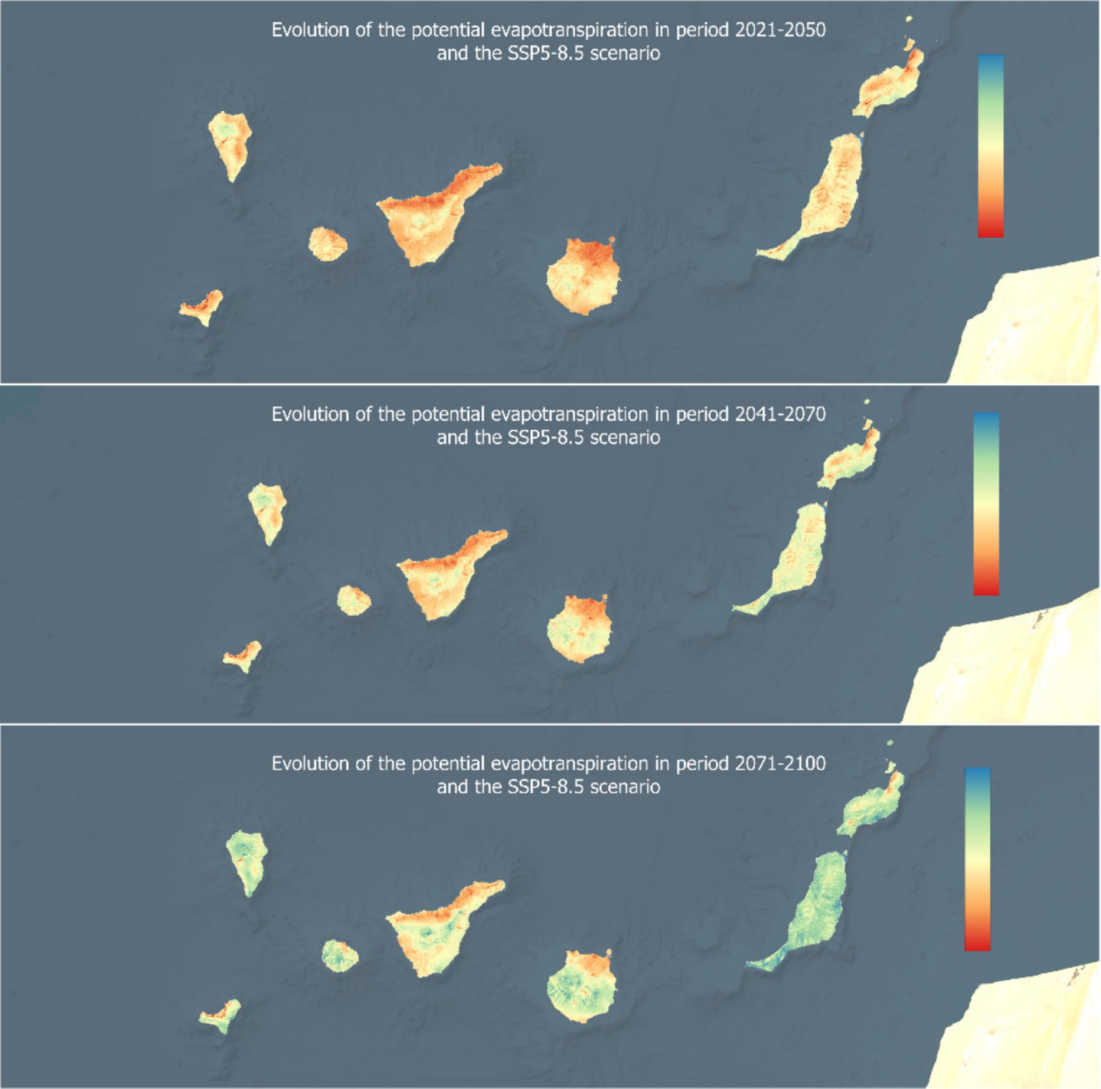
Evolution of the potential evapotranspiration in three time periods for the SSP5-8.5 scenario

**Fig. 7:**
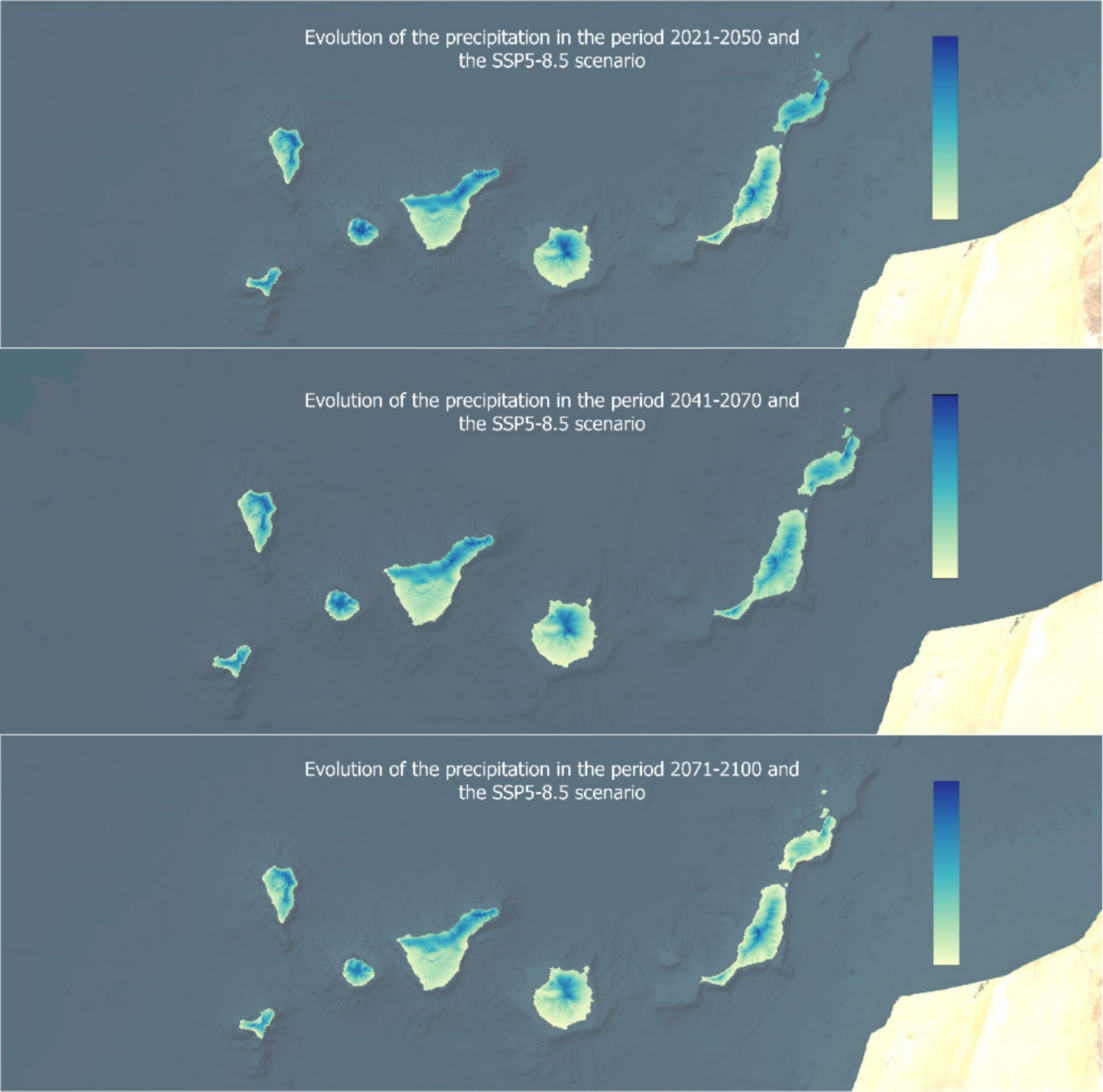
Evolution of the precipitation in three time periods for the SSP5-8.5 scenario

Although each island exhibits distinct characteristics and water patterns, the overall trend points to a declining water balance, worsening as the century progresses due to increased evapotranspiration and stable or decreasing precipitation levels. The two weather patterns are illustrated in Figs. 6 and 7, based on the SSP5-8.5 scenario. Additionally, it is important to note that the water balance in coastal areas of all seven analyzed islands is currently zero, indicating that these regions will face even greater water stress in the long term.

Precipitation plays a fundamental role in the hydrological cycle as the primary source of water for terrestrial ecosystems (Eekhout et al., 2018). Shifts in precipitation patterns directly affect water availability, groundwater recharge, and surface water dynamics, all of which are crucial for maintaining the overall water balance (Eekhout et al., 2018). Climate models from the CMIP6 and the Intergovernmental Panel on Climate Change’s Sixth Assessment Report indicate a global trend of decreasing precipitation, particularly in arid and semi-arid regions but also in tropical and temperate climates.

The extent of precipitation decline varies across regions, with some areas expected to experience significantly greater reductions than others (Benítez et al., 2016). These changes will have profound impacts on regional hydrological cycles, intensifying water stress, increasing evaporation rates, and making droughts more frequent (Djaman et al., 2016; Eekhout et al., 2018). Declining precipitation will directly impact water availability for human consumption, agricultural productivity, and ecosystem sustainability (Eekhout et al., 2018; Puma-Cahua, 2023). Reduced rainfall threatens food security, livelihoods, and industries dependent on water resources (Cruz‐Pérez et al., 2023; Maqbool, 2023). Additionally, shrinking mountain snowpacks, altered river flows, and shifting rainfall patterns further exacerbate hydrological imbalances (Eekhout et al., 2018; Myhre et al., 2018). Lower precipitation levels will heighten social and economic pressures, necessitating coordinated strategies to support both communities and ecosystems (Djaman et al., 2016; Rodríguez-Urrego et al., 2022; Schmitz et al., 2017; Sharma et al., 2018).

Such a general decrease in precipitation (Fig. 7), coupled with an increase in temperature and solar radiation, will lead to an increase in evapotranspiration, reaching severe values by the end of the century. Leta et al. (2018) highlight the significant impact of evapotranspiration on the water balance of volcanic islands such as Hawaii. The results obtained in the present investigation, which show a trend of increasing evapotranspiration and decreasing precipitation in the Canary Islands, agree with previous studies. Although precipitation remains a difficult parameter to project due to its variability and the complexity of the atmospheric processes involved, different studies point to a decrease in rainfall in the Canary Islands (Benítez et al., 2016; Carrillo et al., 2023). Adinolf et al. (2025) establish a decrease in the frequency and average rainfall in Tenerife and Fuerteventura, being more difficult to predict in Tenerife given its complex orography. In addition to the decrease in rainfall in the different islands, a lower soil humidity (Sosa-Guillén et al., 2024) is also predicted, which will influence the availability of the resource. In reference to evapotranspiration, the results also agree with other studies that foresee a future increase of this indicator, higher in the SSP5 scenario (Carrillo et al., 2023).

Given the region’s already scarce water resources, rising temperatures and increased evaporation rates present additional challenges (Cruz‐Pérez et al., 2023). The combined demands of urban expansion, agriculture, and tourism further strain water supplies (Dorta Antequera et al., 2021). Reduced rainfall affects groundwater recharge, stream flows, and the long-term stability of water resources. In response to these pressures, local authorities have implemented desalination, wastewater reuse, and rainwater harvesting to mitigate shortages (Cruz‐Pérez et al., 2022; León et al., 2021; Peñate et al., 2011; Velázquez-Medina, 2023). These strategies diversify water sources and improve efficiency, but additional measures are required. The use of renewable energy in desalination could reduce reliance on fossil fuels, while collaboration with other island regions may offer further adaptive solutions.

The decline in precipitation has severe implications for agriculture and tourism, two of the Canary Islands’ economic pillars and the sectors with the highest water demand. Swift action is necessary to address these challenges. The adoption of water-saving irrigation techniques, drought-resistant crops, and updated agricultural policies could help mitigate the impact on farmers. Similarly, the tourism sector depends on stable water availability, making integrated planning essential (Dorta Antequera et al., 2021; Mendoza-Grimón et al., 2015). By leveraging technological innovation, policy interventions, and community participation, the Canary Islands can enhance resilience to climate-induced water stress and safeguard long-term sustainability (Cruz‐Pérez et al., 2023).

Finally, it is essential to acknowledge the uncertainties and limitations associated with each step of the methodology. Climate models inherently contain uncertainties, as they represent past and future climate states as a single realization. Additionally, the climate system exhibits variability across multiple temporal scales, ranging from atmospheric days to oceanic years. To mitigate these limitations, a 30-year period was selected for the climate analysis. Beyond model- and climate-related variability, the SSPs used to drive future projections constitute plausible trajectories that may result in different climate states, adding an additional layer of complexity and uncertainty to the interpretation of results and their associated local impacts. The primary strategy to address these limitations is the ensemble approach, in which the same model is initialized under slightly different conditions, or multiple models are employed to simulate the same SSP scenario. Both approaches are implemented in CMIP6, providing combinations of models, SSPs, and time horizons to generate coherent projections. This ensemble framework enables the exploration of a range of potential outcomes and impacts under future climate states.

## Conclusions

Water balance measures the relationship between precipitation, desalination, and imported water against evaporation, transpiration, and withdrawals. Maintaining equilibrium between inputs and outputs is essential for sustaining agriculture, promoting growth, and reducing the risks associated with drought. The impacts of climate change pose a serious threat to the availability of essential resources necessary for sustainable development of territories. Global climate projections indicate a rise in temperatures and a decline in precipitation, which will significantly affect water resources, particularly in regions already experiencing high levels of water stress.

The Canary Islands, as a territory highly dependent on rainfall and natural water sources, face critical challenges in ensuring long-term sustainability of their water supply. In addition, human activities and population growth further increase water demand in the archipelago.

Given the increasing pressures on water resources, it is essential to develop forecasting models that assess how hydrological balance will evolve in the coming decades. The FICLIMA methodology has been implemented to analyze these variations, providing a high-resolution representation (100 m) of changes in the water balance across each island throughout the twenty-first century. One of the key strengths of this methodology is its ability to capture the impact of complex topographies and microclimatic variations, offering valuable insights into localized hydrological changes.

The general trend observed in the projections points to a progressively decreasing water balance, exacerbated by increasing evapotranspiration rates and either stable or declining levels of precipitation. The analysis also highlights how these changes are influenced by altitude, with coastal areas exhibiting an almost negligible water balance. In the case of El Hierro, projections indicate a reduction of between 50 and 75% in the island’s hydrological balance by the end of the century. Meanwhile, Gran Canaria is expected to experience an almost complete depletion of its water reserves. The easternmost islands, historically characterized by their arid conditions, already have an extremely low hydrological balance, making their anticipated increase in water stress particularly alarming.

To enhance water resilience in the region, it is necessary to implement an integr`ated and sustainable water management approach. The findings of this study provide useful data for the regulation of water use in the Canary Islands, especially as competition intensifies for this resource from demanding sectors such as tourism and agriculture. Strategies currently in place, including desalination technologies, water recycling systems, and improvements in storage and distribution infrastructure have played a role in mitigating water shortages. However, these approaches continue to face challenges related to efficiency and energy consumption.

By adopting adaptive policies, the Canary Islands can improve their capacity to address future water scarcity and ensure the long-term viability of both natural ecosystems and human activities. In this context, fostering regional cooperation, advancing research in hydrological forecasting, and integrating climate resilience principles into policy frameworks will be essential for securing water resources in a changing climate.

## Data Availability

Data on projections over the century are available on the public access platform SICMA-Canarias (Santamarta et al., 2025).
